# Fibroblasts From Idiopathic Pulmonary Fibrosis Induce Apoptosis and Reduce the Migration Capacity of T Lymphocytes

**DOI:** 10.3389/fimmu.2022.820347

**Published:** 2022-02-10

**Authors:** Leslie Chavez-Galan, Carina Becerril, Andy Ruiz, Lucero A. Ramon-Luing, José Cisneros, Martha Montaño, Alfonso Salgado, Carlos Ramos, Ivette Buendía-Roldán, Annie Pardo, Moisés Selman

**Affiliations:** ^1^ Instituto Nacional de Enfermedades Respiratorias, “Ismael Cosío Villegas”, Mexico City, Mexico; ^2^ Facultad de Ciencias, Universidad Nacional Autónoma de México, Mexico City, Mexico

**Keywords:** idiopathic pulmonary fibrosis, T cells, apoptosis, migration, fibrotic focus

## Abstract

Idiopathic pulmonary fibrosis (IPF) is a progressive and irreversible lung disease of unknown etiology. Myofibroblasts are organized in peculiar subepithelial fibroblasts foci (FF), where they abnormally persist and exclude lymphocytes by unclear mechanisms. FF are the source of an excessive extracellular matrix, which results in progressive stiffening and destruction of the lung architecture. We hypothesized that the absence of T cells inside the FF could be related, at least partially, to an inefficient function of lymphocytes induced by IPF fibroblasts. Here, we evaluated the effect of a supernatant from IPF fibroblasts on T-cell apoptosis and migration capacity. Data showed that IPF fibroblasts secrete pro-apoptotic molecules (both from extrinsic and intrinsic pathways), generating a microenvironment that induces apoptosis of T cells at 3 h of culture, despite a weak anti-apoptotic profile exhibited by these T cells. At 24 h of culture, the supernatants from both IPF and control fibroblasts provoked T-cell death. However, at this time of culture, IPF fibroblasts caused a marked decrease in T-cell migration; in contrast, control lung fibroblasts induced an increase of T-cell migration. The reduction of T-cell migratory capacity provoked by IPF fibroblasts was associated with a negative regulation of RHOA and ROCK, two essential GTPases for migration, and was independent of the expression of chemokine receptors. In conclusion, our findings demonstrate that IPF fibroblasts/myofibroblasts induce apoptosis and affect T-cell migration, revealing a mechanism involved in the virtual absence of T lymphocytes inside the FF.

## Introduction

Idiopathic pulmonary fibrosis (IPF) is a specific form of chronic, progressive, fibrosing interstitial pneumonia of unknown etiology that occurs primarily in older adults, and is characterized by the histopathologic and/or radiologic pattern of usual interstitial pneumonia (UIP) (1, 2). The pathogenic mechanisms are uncertain, but strong evidence indicates that the early and critical event is the aberrant activation of lung epithelial cells which undergo strong phenotypic and functional changes inducing a progressive and multistep process that involves fibroblast activation, extracellular matrix remodeling, and finally, the fibrotic destruction of the lung architecture (2–5).

In this sequence, IPF fibroblasts undergo strong activation and transdifferentiate in myofibroblasts leading to a chaotic production of large amounts of extracellular matrix components mainly fibrillar collagens (2, 3, 5, 6).

Interestingly, in IPF, interstitial spindle-shaped fibroblasts and myofibroblasts organize in the so-called fibroblastic foci (FF), represented by small dome-shaped collections of cells within a myxoid-appearing matrix with their surface covered by hyperplastic alveolar lining cells (7). FF are usually arranged with their long axis parallel to the long axis of the alveolar septa, and it has been considered to represent a leading edge of fibrogenesis during the development of IPF (7–9).

It has been suggested that unlike normal granulation tissue resolution, where fibroblasts/myofibroblasts undergo apoptosis and are removed during repair (10), in IPF, these cells persist probably by increasing resistance to apoptosis likely associated with a senescent phenotype (11, 12). By contrast, in fibroblasts from Masson bodies in organizing pneumonia, an intra-alveolar structure similar to FF usually resolves almost completely, and fibroblasts are eliminated by increased apoptotic activity (13).

**Figure 1:**
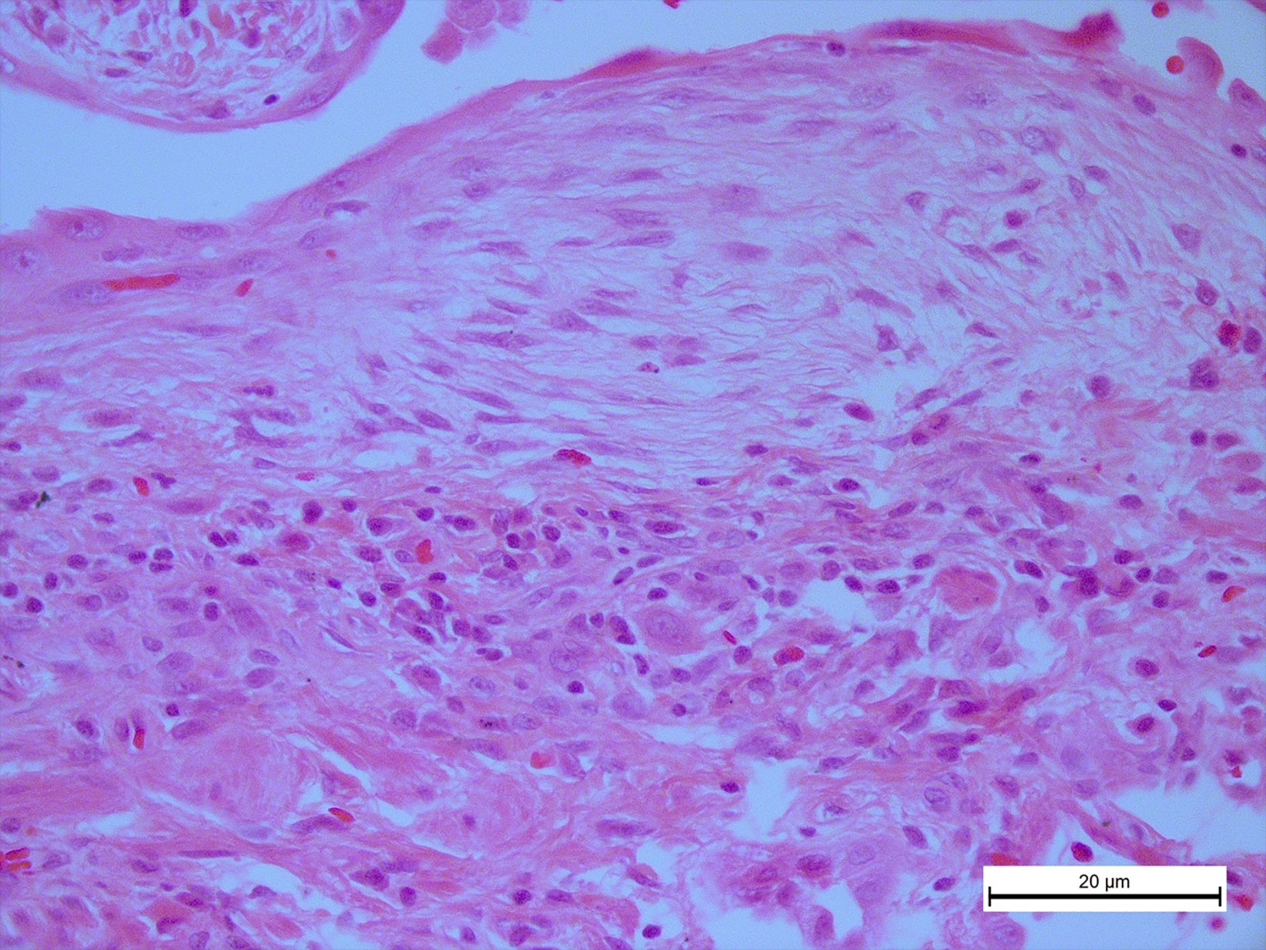
In idiopathic pulmonary fibrosis, T cells are virtually absent inside the fibroblastic foci. A photomicrograph representative from the lung tissue of an idiopathic pulmonary fibrosis (IPF) patient stained with hematoxylin and eosin showing numerous inflammatory cells, primarily lymphocytes around a typical fibroblastic focus, original magnification ×40.

In this scenario, although there is some background of usually a mild degree of chronic inflammation, represented mostly by lymphocytes, these are virtually absent inside the fibroblastic focus (**Figure 1**) (7). This finding could represent an important biopathological process contributing to the persistence of fibroblast since T cells play a relevant role in the clearance of senescent cells, including lung fibroblasts (14).

In this context, we hypothesized that the persistence of fibroblasts/myofibroblasts in this disease could be provoked by an aggressive behavior of these mesenchymal cells inducing apoptosis and/or reducing the migration of T lymphocytes, avoiding the elimination of the fibroblasts/myofibroblasts by the immune system.

## Materials and Methods

### Human Material

The research protocol was approved by the Ethics Committee of Instituto Nacional de Enfermedades Respiratorias Ismael Cosio Villegas (# B33-20). Healthy donors provided written informed consent to allow the use of their blood samples for research.

### Fibroblast Isolation and Culture

Fibroblast primary cell lines from normal lungs (control human lung fibroblasts, CLF) and IPF lungs were obtained in our laboratory as described and used within five to nine passages of the initial isolation (15, 16). Briefly, primary cultures were grown in Ham’s F-12 medium (Gibco Laboratories, Grand Island, NY, USA) supplemented, and cell cultures were maintained at 37°C and 5% CO_2_.

The supernatants used in this study were obtained from lung fibroblasts of 10 different IPF patients [mean age 66 years (min 57, max 81), 90% men] and four healthy donors [mean age 61 years (min 53, max 69), all men]. IPF patients were not receiving anti-fibrotic therapy before biopsy.

### Cell Supernatants

CLF and IPF human lung fibroblasts were seeded, and at 24 h of culture, the supernatant was collected and stored at −80°C until use. Hereafter, the supernatant recovered from IPF fibroblasts is called “IPF (SN)” and the supernatant recovered from control human lung fibroblasts is called “CLF (SN)”.

### T-Cell Enrichment

Peripheral blood mononuclear cells (PBMC) were obtained from healthy donors. Total T cells were enriched using negative selection (Pan T Cell Isolation Kit, Miltenyi Biotech Bergisch Gladbach, Germany). Briefly, after delimiting live and singlet cell gates in the enriched CD3+ fraction, the expression of CD3, CD19, and CD14 was evaluated to remove the contamination of B cells (CD19+) and monocytes (CD14+). We also used the coexpression of CD2 in the CD3+ cells to further confirm the lymphocyte origin of these T cells (**Figure S1A**). The purified cells were routinely 93% to 97% of the intended cell type by flow cytometry.

### T-Cell Culture

T cells were plated (1 × 10^6^ cells/well) in 24-well plates (Costar, ON, Canada) with 1 ml of a) RPMI 1640 medium (Gibco, Grand Island, NY, USA), supplemented, or 1 ml of b) IPF (SN) and c) CLF (SN). The cell culture was conducted for 3 and 24 h at 37°C and 5% CO_2_.

### Flow Cytometry

Enriched T cells were harvested and stained with mAb to CD2, CD3, CD4, CD8, CD14, CD19, chemokine C-C motif receptor-2 (CCR2) and -7 (CCR7), C-X-C motif chemokine receptor-1 (CXCR1) and -4 (CXCR4), TNF, TNFR1, TNFR2, CD28, KLRG1, and PD-1 (BioLegend, San Diego, CA, USA). Multiparametric flow cytometry was performed using a FACS Aria II flow cytometer (Becton Dickinson, San Jose, CA, USA). Fluorescence minus one (FMO) controls were stained and acquired in parallel. Typically, 100,000 events were recorded. Data were analyzed with the FlowJo software (Tree Star, San Carlos, CA, USA).

### Evaluation of Cell Death by Flow Cytometry

T cells were harvested (ending the culture) and stained with mAb to CD4 and CD8, posteriorly washed, and suspended in staining buffer (BioLegend) with 3 μl 7-AAD solution for 20 min at 4°C in the dark. Then, the cells were washed and suspended in binding buffer 1× with 5 μl Annexin-V Brilliant Violet 421-conjugated (BioLegend). T cells cultured with staurosporine (STU) 1 μg/ml (Sigma-Aldrich, St. Louis, MO, USA) were used as a positive control of apoptosis.

Cells were acquired using a FACS Aria II flow cytometer (Becton Dickinson). Typically, 100,000 events per sample were recorded. Data were analyzed with the FlowJo software.

### RNA Extraction and Reverse Transcription

The total RNA of T cells was obtained using RNeasy Micro Kit (Qiagen, Hilden, Germany) according to the instructions of the manufacturer. RNA amount was evaluated by the Qubit™ assay kit in the Qubit 2.0 Fluorometer (Life Technologies, Waltham, USA). A total of 112.50 ng of total RNA was converted to cDNA using High-Capacity cDNA Reverse Transcription Kit (Applied Biosystems, Waltham, USA) in a volume of 30 μl following the guidelines of the manufacturer.

### Quantitative Polymerase Chain Reaction

Quantitative real-time PCR was performed using TaqMan probes specific for the following: Ras homolog family member A (RHOA, Hs00357608_m1), Unconventional myosin-IXb (MYO9B, Hs00188109_m1), Rho-associated coiled-coil-containing protein kinase 1 (ROCK1, Hs01127701_m1), BH3 interacting domain death agonist (BID, Hs00609632_m1), Direct inhibitor of apoptosis-binding protein with low p*I* (DIABLO, Hs00219876_m1), B-cell lymphoma 2 (Bcl-2, Hs04986394_s1), myeloid cell leukemia 1 (Mcl-1, Hs06626047_g1), tumor necrosis factor receptor 1 (TNFR1, Hs01042313_m1), tumor necrosis factor receptor 2 (TNFR2, Hs00961750_m1), ACTB (β-actin) (Hs01060665_g1), and 18S (18S ribosomal RNA gene) (Hs03928990_g1). Single reactions were prepared with the Maxima Probe/ROX qPCR Master Mix (Thermo Fisher Scientific, Waltham, USA), and all amplifications were run in duplicate under the following thermal conditions: 95°C for 10 min followed by 40 cycles of 60°C for 1 min and 95°C for 15 s, with the StepOnePlus™ Real-Time PCR Systems (Applied Biosystems). The relative expression of transcripts was quantified using the ΔΔCT method. The *n*-fold change was calculated for each gene, normalizing to the endogenous controls ACTB and 18S and relative to a control group without stimulus during the culture of T cells from four healthy donors (=1). Then, the log2 (fold change) in CLF and IPF was determined, where log2 (fold change) of control condition = 0.

### Profile of Apoptosis-Related Proteins in Fibroblast Supernatant

We evaluated 35 molecules related to apoptosis in two biological replicates, using two independent supernatants from CLF and two from IPF. Proteins were quantified (Bradford assay) and maintained at −80°C until use. The profile of apoptosis-related proteins was evaluated using the Proteome Profiler™ kit (R&D Systems, Minneapolis, MN, USA). Briefly, 360 μg/protein diluted in 1.5 ml of array buffer (total volume) was added to the membrane and incubated overnight, and posteriorly, a detection antibody cocktail was added. Finally, protein spots were detected and visualized with the Imaging System from Bio-Rad (ChemiDoc™ XRS+ System). Pixel density was analyzed by densitometry using the online ImageJ 1.39c software provided by NIH.

### Cell Migration Assay

Migration T-cell assays were performed based on previous standardization assays and conditions reported for T-cell migration (17). Chemoattractants CCL2 (MCP-1) (200 ng/ml) and CCL19 (200 ng/ml) (PeproTech Cranbury, NJ; USA) in 150 μl of RPMI media were added alone or together to the bottom well of a 96-well chemotaxis plate (Cytoselect 96-well cell migration assay-fluorometric format, Cell Biolabs, Inc. San Diego, CA, USA.). T cells cultured 24 h with supernatants were recovered and suspended in RPMI 1640 serum-free media (4 × 10^6^ cells/ml), and 100 μl of suspension cells were added on top of the membrane (5 μm pore size). Cells were allowed to migrate for 3 h at 37°C in 5% CO_2_. Cells in the membrane were recovered using 150 μl of cell detachment solution and lysed. The total number of transmigrated cells was quantified using CyQuant^®^ GR Dye (Synergy™ HT, BioTek, Inc., USA). Cells in RPMI 1640 with BSA 1% were used as blanks for each chemokine.

### Flow Cytometric Bead Array Analysis

The levels of interleukin (IL)‐1β, IL‐6, IL‐10, and IL‐12p70 were measured in the supernatants obtained from T cells, IPF, and control lung fibroblasts using a flow cytometric bead array (CBA) kit (Becton Dickinson) according to the protocol of the manufacturer. Briefly, standard cocktail and samples were prepared with assay buffer and bead cocktails. Then, tubes were incubated 2 h at room temperature protected from the light. Detection antibodies were added to each tube and incubated at room temperature for 1.5 h. Samples were analyzed with a FACS Aria II flow cytometer (Becton Dickinson), and the concentrations of cytokines were calculated using the FCAP Array software, following the instructions of the provider (Becton Dickinson).

### ELISA Sandwich Assays

IL-2 (Cat. No. 431805, BioLegend) and CCL2 (Cat. No. 438805, BioLegend) were quantified in the supernatants obtained from T cells, IPF, and control lung fibroblasts by an ELISA assay according to the instructions of the manufacturer.

### Western Blot

T cells were lysed and suspended in Laemmli buffer. SDS-PAGE was performed under non-reducing conditions, and proteins were transferred to a 0.2-μm pore size polyvinylidene difluoride (PDFV) membrane (Bio-Rad, Hercules, CA, USA). Western blot was made using the following antibodies: anti-Mcl-1 (dilution 1:1,000; Cell Signaling Technology, Danvers, MA, USA), anti-DIABLO (dilution 1:1,000; Cell Signaling Technology), anti-glyceraldehyde 3-phospate dehydrogenase (GAPDH, dilution 1:1,000; GeneTex Biotechnology, Irvine, CA, USA), and anti-mouse- and anti-rabbit horseradish peroxidase-labeled IgG (both used 1:2,000; R&D Systems, Minneapolis, MN, USA). Protein bands were detected by incubation with enhanced chemiluminescence reagent (Thermo Scientific, Pierce Biotech., Rockford, IL, USA) and visualized with the Imaging System from Bio-Rad (ChemiDoc™ XRS+ System). Band densities were analyzed by densitometry using ImageLab 6.0.1 software provided by Bio-Rad. Each sample was normalized using GAPDH as a loading control.

### Statistical Analysis

Data represent mean ± standard deviation (SD) for each group. Database, statistics, and graphics were made with GraphPad Prism 9 (GraphPad Software, USA). We performed a multiple *t*-test and Holm–Sidak as post-test for multiple comparisons and a Mann–Whitney *U* test to compare between two groups.

## Results

### Supernatant From IPF Fibroblasts Increases T-Cell Death

**Figure 2:**
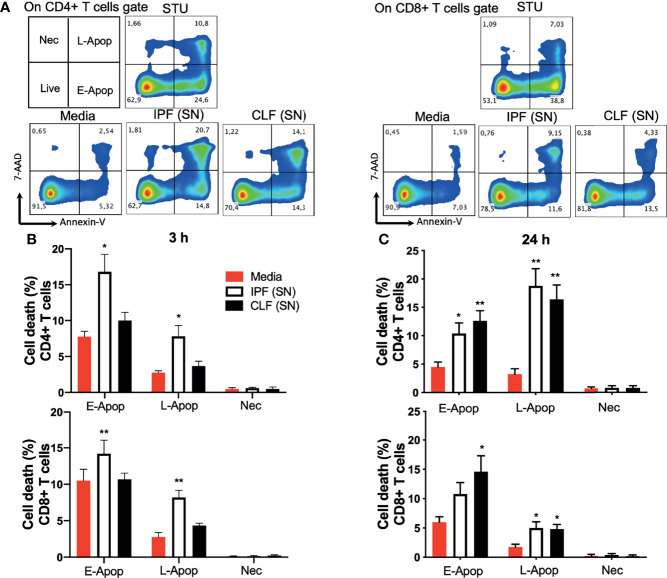
The supernatant (SN) from IPF fibroblasts increases T-cell death at short-time exposure. T cells from five healthy donors were cultured during 3 and 24 h with different IPF (SN) or control lung fibroblasts SN. T cells cultured with media were used as a negative control and those cultured with 1 μg/ml staurosporine (STU) were used as a positive control of apoptosis. **(A)** CD4+ T-cell and CD8+ T-cell gates were delimited, and cell death was analyzed in a dot plot color where Annexin-V+ cells indicated early apoptosis (E-Apop), Annexin-V+7-AAD+ late apoptosis (L-Apop), and 7-AAD+ necrotic cells (Nec). **(B)** Percentage of cell death evaluated at 3 h of culture. **(C)** Percentage of cell death evaluated at 24 h of culture. Bars indicate mean ± SD from the five independent biological experiments. A multiple *t*-test and Holm–Sidak as post-test were used. *p < 0.05, **p < 0.01.

By flow cytometry, we assessed early and late apoptosis and necrosis of T cells from healthy donors, cultured during 3 and 24 h with IPF (SN) or CLF (SN), media alone was used as a negative control. First, gates of CD4+ T cells and CD8+ T cells were delimited (**Figure S1B**), and cell death was analyzed in a dot plot color where Annexin-V+ cells indicated early apoptosis (E-Apop), Annexin-V+7-AAD+ late apoptosis (L-Apop), and 7-AAD+ necrotic cells (Nec) (**Figure 2A**).

Our data showed that compared with T cells cultured with medium, IPF (SN) increases cell death of both CD4+ and CD8+ T cells at 3 h of culture, while CLF (SN) showed no effect. In CD4+ T cells stimulated with IPF (SN), E-Apop displayed a two-fold increase (16.8% versus 7.7%), while L-Apop exhibited a three-fold increase (7.8% versus 2.5%) (**Figure 2B**, up). Similarly, IPF (SN) increased E-Apop (14.2% versus 10%) and L-Apop (8.2% versus 2.7%) in CD8+ T cells (**Figure 2B**, down). In contrast, at a longer time (24 h), the supernatants of both IPF and CLF increased E-Apop and L-Apop of CD4+ and CD8+ T cells (**Figure 2C**, up and down).

These data support our hypothesis that IPF (SN) induces cell death of T cells after a short-time exposure (3 h), although at 24 h, both IPF and CLF supernatants similarly increase T-cell death.

### IPF Fibroblasts Secrete High Levels of Pro-Apoptotic Molecules

**Figure 3:**
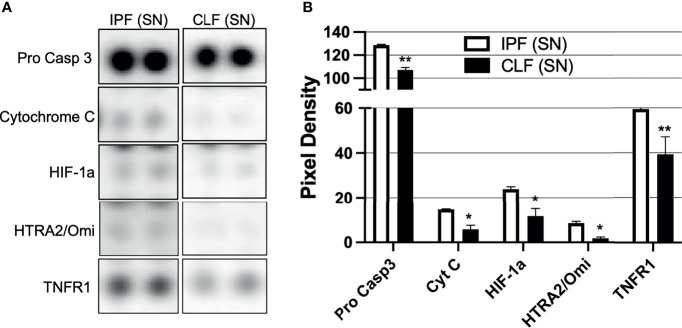
The supernatant (SN) from IPF fibroblasts promotes a pro-apoptotic microenvironment. Fibroblast supernatants from two different IPF lungs and two healthy donors were recovered until having 360 μg/protein, then a Proteome Profiler™ kit was used. **(A)** Representative membrane for pro-caspase 3, cytochrome C, HIF-1a, HTRA2/Omi, and TNFR1. **(B)** Pixel density was obtained by densitometry using online ImageJ 1.39c software. Bars indicate mean ± SD from two independent experiments (two independent supernatants from IPF and CLF); each molecule was evaluated in duplicate. A Mann–Whitney *U* test was used. *p < 0.05, **p < 0.01.

To identify putative molecules involved in T-cell death induced by the IPF (SN) at 3 h of culture, we performed a human apoptosis array in both IPF (SN) and CLF (SN) and evaluated 35 apoptosis-related proteins (**Figure S2**). In **Figure 3**, we summarized the profile of pro-apoptotic molecules that were increased in the IPF (SN) compared with CLF (SN) (**Figure 3A**). As illustrated in **Figure 3B**, IPF (SN) has increased the level of the pro-apoptotic molecules: pro-caspase 3, cytochrome C, hypoxia-inducible factor 1 alpha (HIF-1a), the high-temperature requirement 2 (HTRA-2/Omi), and the tumor necrosis factor receptor 1 (TNFR1).

Together, our data suggest that T-cell apoptosis observed after a short-time exposure (3 h) to IPF (SN) could be a consequence of the high level of this pro-apoptotic profile that is released by the fibroblasts from IPF.

### Short Time of IPF (SN) Exposure Affects the Anti-Apoptotic/Pro-Apoptotic Profile of T Cells

**Figure 4:**
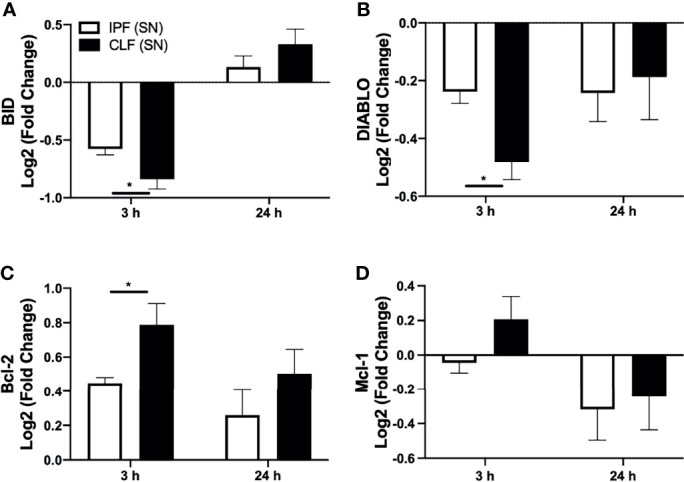
The supernatant (SN) from IPF fibroblasts affects the Bcl-2 anti-apoptotic profile of T cells at a short time of exposure. T cells from five different healthy controls were stimulated with different IPF (SN) and CLF (SN), recovered at the end of 3 and 24 h of culture, and RNA was extracted for quantitative real-time PCR. We calculated the log2 fold change of the expression of BID **(A)**, DIABLO **(B)**, Bcl-2 **(C)**, and Mcl-1 **(D)**. Bars indicate mean ± SD from five independent biological experiments. A Mann–Whitney *U* test was used. *p < 0.05.

We were interested to clarify if the pro-apoptotic stimulus provided by IPF (SN) modifies the apoptotic profile in T cells at 3 h of culture. In this context, the transcriptional levels of pro-apoptotic (BID and DIABLO) and anti-apoptotic (Bcl-2 and Mcl-1), which are related to the intrinsic pathway of apoptosis, as well as TNFR1, which is involved in the extrinsic pathway, were evaluated in T cells after 3 and 24 h of culture with IPF (SN) and CLF (SN) (**Figure 4**).

Our results indicated that at 3 h, T cells stimulated with either IPF (SN) or CLF (SN) showed a significant decrease in the expression of pro-apoptotic genes BID and DIABLO; however, T cells exposed to IPF (SN) showed a less dramatic downregulation compared with those induced by CLF (SN). By contrast, at 24 h of exposure, both supernatants induce a similar profile of BID and DIABLO (**Figures 4A, B** **)**. Interestingly, both IPF (SN) and CLF (SN) induced in T cells a positive regulation of the anti-apoptotic molecule Bcl-2 at 3 and 24 h (**Figure 4C**), though at 3 h of culture, T cells/IPF (SN) displayed a lower expression of Bcl-2 than T cells/CLF (SN). Moreover, at 3 h of culture, T cells/IPF (SN) regulated negatively Mcl-1, whereas those T cells/CLF (SN) induced Mcl-1 (**Figure 4D**).

**Figure 5:**
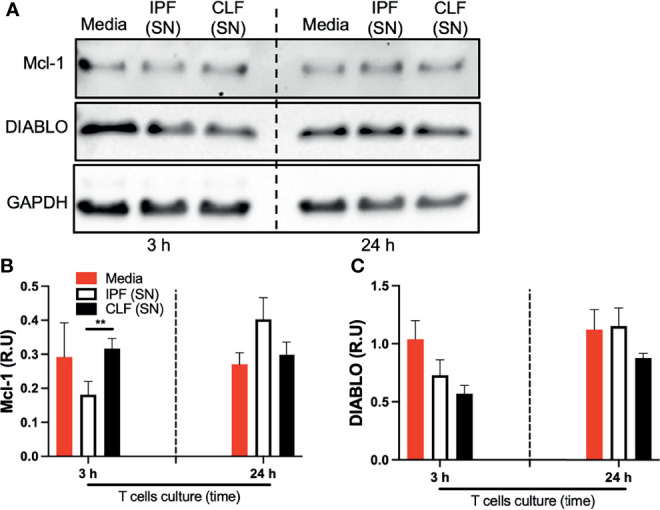
The supernatant (SN) from IPF fibroblasts decreases Mcl-1 at the protein level in T cells at a short time of exposure. T cells obtained from four different healthy donors were stimulated with different IPF (SN) and CLF (SN) and recovered at the end of the culture. Then, Mcl-1 and DIABLO were examined by Western blot **(A)**. Band densities were normalized with GAPDH by densitometry analysis and results are shown in relative units (RU) of Mcl-1 and DIABLO concentration (**B, C**, respectively) using ImageJ software. Bars indicate mean ± SD from four independent biological experiments. A multiple *t*-test and Holm–Sidak as post-test were used. **p < 0.01.

For further confirmation, we evaluated DIABLO and Mcl-1 at the protein level by Western blot (**Figure 5A**). Interestingly, at the protein level, the anti-apoptotic Mcl-1 was strongly downregulated by IPF (SN) at short-time exposure (**Figure 5B**). Although no differences were observed with the levels of DIABLO, IPF and control supernatants reduced the expression of this apoptotic protein at 3 h of culture (as we observed with RT-PCR) (**Figure 5C**).

These findings suggest that T cells exposed to the fibroblast supernatant activate an anti-apoptotic profile as a defense mechanism to the received stimuli, characterized by the downregulation of pro-apoptotic and upregulation of anti-apoptotic Bcl-2 family members. However, this response is less efficient when stimulated with IPF (SN).

### Short-Time Exposure to IPF (SN) Induces the Expression of tmTNF and a Positive Regulation of TNFR1 in T Cells

**Figure 6:**
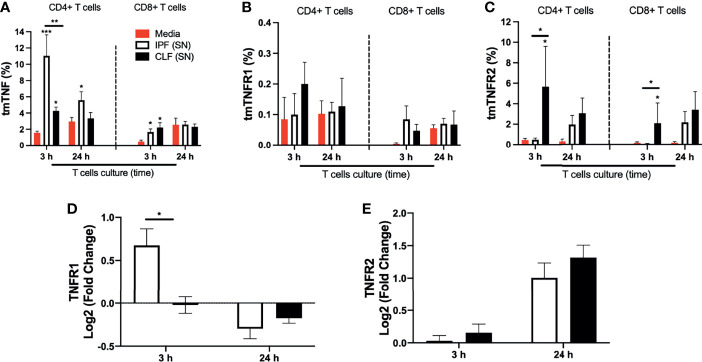
The supernatant (SN) from IPF fibroblasts increases tmTNF expression and positively regulates TNFR1 at the transcriptional level in T cells at a short time of exposure. T cells obtained from five different healthy donors stimulated with different IPF (SN) and CLF (SN) were recovered at the end of the culture, and cells were prepared for flow cytometry and for obtention of RNA to perform a quantitative real-time PCR. The percentages of CD4+ T cells (left) and CD8+ T cells (right) positive to transmembrane (tm) TNF **(A)**, tmTNFR1 **(B)**, and tmTNFR2 **(C)** were obtained by flow cytometry. To acquire the transcriptional level, we calculated the log2 fold change of TNFR1 **(D)** and TNFR2 **(E)**. Bars indicate mean ± SD from five independent biological experiments. A multiple *t*-test and Holm–Sidak as post-test **(A–C)** or a Mann–Whitney *U* test **(D, E)** was used. *p < 0.05, **p < 0.01, ***p < 0.001.

IPF (SN) secreted increased levels of TNFR1, which could interact with the transmembrane TNF to regulate the TNFRs and TNF production. To clarify if the transmembrane (tm) forms are induced by IPF (SN), these molecules were evaluated by flow cytometry (**Figure S3**). Data showed that IPF (SN) increased the expression of tmTNF on CD4+ and CD8+ T cells at 3 h (mean 11% and 2%, respectively) compared with CD4+ and CD8+ T cells in media (mean 1.8% and 0.3%, respectively), and the CD4+tmTNF+ was maintained even at 24 h of culture (mean 5%). CLF (SN) also increased the frequency of CD4+tmTNF+ and CD8+tmTNF+ at 3 h (mean 4% and 2.3%, respectively), but it was lower than the level induced by IPF (SN) (**Figure 6A**). Although the frequency of T cells positive to tmTNFR1 was not different at 3 and 24 h of culture (**Figure 6B**), CFL (SN) strongly increased the frequency of T cells positive to tmTNFR2 at 3 h of culture (**Figure 6C**).

As a further confirmation, we observed that at the transcriptional level, IPF (SN) induced a positive regulation of TNFR1 in T cells at 3 h, whereas CLF (SN) induced a negative regulation (**Figure 6D**), and at 24 h, both supernatants provoked a negative regulation. Our result also showed that TNFR2, a receptor not involved in apoptosis, is positively regulated with both SN (**Figure 6E**). The results at the transcriptional level were normalized with the value obtained with T cells cultured with media alone.

Finally, we confirmed that although IPF (SN) and CLF (SN) cause a different expression of the TNF pathway molecules, there are no changes in cytokine production by T cells, suggesting that these cells are not activated nor show a pattern of an exhausted phenotype (**Figure S4**).

Together, these data suggest that the TNFR1 level present in the IPF (SN) induces the expression of tmTNF, and likely, this axis contributes to T-cell apoptosis at 3 h of culture. Although CFL (SN) also induces a discrete increase of the tmTNF, the tmTNFR2 anti-apoptotic signal also is activated in this condition, likely contributing to T-cell survival.

### IPF (SN) Decreases the Migratory Ability of T Cells

Since T cells virtually do not enter into the fibroblastic foci, we examined also the possibility that IPF (SN) affects their migratory capacity. For this purpose, T cells were cultured 24 h with either IPF (SN) or CLF (SN). Then, they were recovered, and their migration ability was analyzed using chemokines CCL2 and CCL19, which are important mediators of T-cell migration (18, 19). We selected 24 h of culture because at this time both supernatants induced a similar level of cell death and the cell viability was similar. The early time (3 h) was discarded to avoid an overestimation of the results given that, at this time, only IPF (SN) induced cell death.

**Figure 7:**
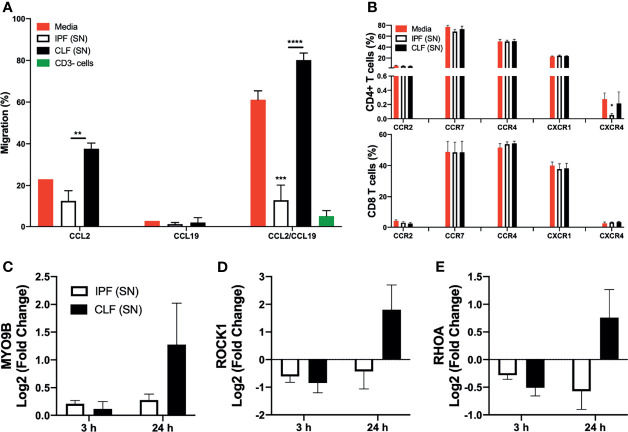
The supernatant (SN) from IPF fibroblasts decreases the migration of T cells at long time of exposure. T cells from five different healthy donors stimulated with different IPF (SN) and CLF (SN) were cultured during 24 h with IPF (SN) or control lung fibroblasts SN, and then cells were recovered to evaluate cell migration and expression of chemokine receptors by flow cytometry. T cells cultured during 3 and 24 h were used to evaluate MYO9B, ROCK1, and RHOA at the transcriptional level. **(A)** T cells were recovered at 24 h and their ability to migrate in response to CCL2, CCL19, and CCL-2/CCL19 was evaluated using a fluorometric assay. The obtained relative fluorescence unit is indicative of the magnitude of cellular migration, and the percentage is obtained normalizing the maximum level of T-cell migration (100%) in response to fetal bovine serum. The CD3− cell fraction (green bar) was used as a negative control of migration to chemokines used. **(B)** Percentage of CD4+ T cells (up) and CD8+ T cells (down) positive to receptors CCR2, CCR7, CCR4, CXCR1, and CXCR4 obtained by flow cytometry. T cells cultured during 3 and 24 h were recovered, and we calculated the log2 fold change of MYO9B **(C)**, ROCK1 **(D)**, and RHOA **(E)**. Bars indicate mean ± SD from five independent experiments. A multiple *t*-test and Holm–Sidak as post-test were used. *p < 0.05, **p < 0.01, ***p < 0.001, ****p < 0.00001.

We found that after 24 h of exposure to IPF (SN), T cells display a significant decrease in migration. In response to CCL2, these cells migrate 60% lesser than those cells cultured with CLF (SN) (12.5% versus 37.6%). Likewise, in response to CCL2+CCL19, IPF (SN) induce a five-fold decrease in migration compared with T cells cultured in culture medium (12.9% versus 61.1%) and a 6-fold decrease compared with T cells cultured with CLF (SN) (12.9% versus 80.1%) (**Figure 7A**). In contrast, T cells cultured with CLF (SN) showed a higher ability to migrate compared with T cells cultured with media, in response to the combination of CCL2/CCL19 (80.1% versus 61.1%; *p* < 0.01) (**Figure 7A**).

No effect was observed using CC19 alone. To confirm the relevance of these cytokines to T-cell migration, the CD3− cell fraction was used and it was unable to migrate in response to CCL2/CCL19 stimuli (**Figure 7A**, green bar). Interestingly, the fibroblast from IPF or CLF produces heterogeneous but not significative different levels of CCL2 (**Figure S5**), suggesting that a deficiency in the secretion of this chemokine is not contributing to the inhibition of T-cell migration.

Since the decrease of migration could be associated with the loss of chemokine receptors in T cells, we evaluated the expression of the receptors to the chemokines used in this experiment (CCR2 and CCR7, respectively), as well as other receptors such as CCR4, CXCR1, and CXCR4. Our data showed that none of them was affected, except CXCR4 expression on CD4+ T cells, but it is important to note that the frequency of these cells is extremely low (less than 0.5%) (**Figure 7B**, up and down). Together, these data suggest that IPF (SN) affects the migratory ability of T cells but without altering the expression of the receptors.

### IPF (SN) Inhibits the Expression of Essential GTPases to Cell Migration

The family of small GTPases is pivotal to induce the migration of T cells; for example, RHOA acts as a molecular switch to bind chemokines, and ROCK regulates the activation of cytoskeletal proteins, and as a complex, RHOA/ROCK is necessary to form microtubules, whereas MYO9B is an activator of RHOA (20).

We evaluated RHOA, ROCK1, and MYO9B at the transcriptional level in T cells cultured 3 and 24 h with IPF (SN) and CLF (SN), and the results were normalized with the value obtained with T-cell cultured with media alone. We found that T cells cultured with both IPF and CLF supernatants did not affect the transcriptional levels of MYO9B, ROCK1, and RHOA at the early time of exposure (**Figures 7C–E**, respectively). In sharp contrast, at 24 h of culture, IPF (SN) induced a negative regulation of ROCK1 and RHOA, whereas CLF (SN) induced a positive regulation of the same genes. Likewise, IPF (SN) induced a weaker expression of MYO9B compared with CLF (SN) (**Figures 7C–E**, respectively).

These results suggest that IPF (SN) affects the migratory capacity of T cells after a long time of stimuli (24 h in culture) affecting the expression of crucial molecules to activate the migration process.

## Discussion

IPF is a chronic, progressive fibrotic lung disease with limited therapeutic options. Myofibroblasts play a critical effector role, inducing prolonged and excessive matrix accumulation and remodeling, leading to progressive lung stiffening and destruction. In turn, matrix stiffness through mechanotransduction pathways also contributes to fibroblast-to-myofibroblast differentiation perpetuating the fibrotic response.

Importantly, while myofibroblasts are transiently detected during normal wound healing, they are persistent/long-lasting in IPF lungs, and this persistence has been related to a lack of removal by apoptosis (10–12, 21).

Several mechanisms intrinsic to the myofibroblast phenotype have been suggested as involved in the resistance to apoptosis including the epigenetic silencing of pro-apoptotic molecules (22). Additionally, strong evidence indicates that fibroblasts/myofibroblasts from IPF lungs are senescent, which are characterized by irreversible cell-cycle arrest, higher resistance to apoptosis, and secretion of a complex set of mediators, known as the senescence-associated secretory phenotype (11, 12, 23).

However, at present, it is unclear whether the disrupted IPF microenvironment favored by fibroblasts affects the neighboring immune cells. In this context, we and others described several years ago that IPF fibroblasts provoked the apoptosis of alveolar epithelial cells *in vivo* and *in vitro* and that this process was a critical factor in the pathogenesis of the disease (11, 24–26). Thus, we hypothesized that a pro-apoptotic environment created by IPF fibroblasts could induce apoptosis of lymphoid cells, affecting the activation of an adaptative immune response and contributing to their persistence in the fibroblastic foci.

Interestingly, reports suggest that myofibroblasts from the lungs with active and progressive fibrosis may acquire an “immune-privilege-like” phenotype resulting in their unrelenting accumulation associated, at least partially, to an inefficient function of the immune system (27, 28). In this setting, there is evidence indicating that T cells may have a role in the removal of fibroblasts; moreover, it has been shown that murine lung myofibroblasts undergo T-cell-induced apoptosis both *in vitro* and during resolution of experimental fibrosis *in vivo* (14, 29–31). In consonance, the resolution of fibrosis and myofibroblast clearance from the lungs of bleomycin-injured mice correlated with their susceptibility to T-cell-induced apoptosis (14). Outstandingly, the apoptotic effect is not observed when the conditioned media of T cells is used, thus excluding the possible effect of secreted factors and supporting that a direct contact between the two cell types is necessary for the induction of fibroblast apoptosis (14).

Our results support the notion that IPF fibroblasts hinder the function of T lymphocytes. Thus, IPF fibroblasts induced T-cell death as early as 3 h of culture, while control lung fibroblasts required more prolonged culture (24 h) to induce a similar percentage of T-cell death observed with IPF fibroblasts. At this time, IPF fibroblasts strongly decrease the migration capacity of T cells.

Importantly, the apoptotic effect of fibroblasts was observed on both CD4+ and CD8+ T cells. We also found that, at least partially, this pro-apoptotic effect was provoked by the secretion of several pro-apoptotic molecules by IPF fibroblasts, and although there is a defensive anti-apoptotic response, this is overcome by the effect of IPF supernatants.

Additionally, short-time exposure to IPF (SN) induced in T cells an increase of the expression of TNFR1, suggesting that TNFR1 could be an alternative way to activate T-cell death. Moreover, evidence supports that the TNF/TNFRs axis induces a positive feedback signaling to self-regulate their presence (32). In this context, our results reveal that the supernatant of IPF fibroblasts stimulates the expression of tmTNF in T lymphocytes suggesting that this pro-apoptotic pathway strongly contributes to the death of these cells.

Another important finding of our study is that T cells recovered at 24 h of exposure to IPF (SN) showing a similar percentage of apoptosis with those cultured with CLF (SN) have strongly affected their migratory capacity in response to different chemokines, and this effect is not associated to changes in the expression of chemokine receptors on the T cell.

The lack of migration ability in T cells exposed to IPF (SN) was associated with the decrease of critical factors that participate in the migration process—RHOA, ROCK, and MYO9B. RHOA is activated when binding to guanosine triphosphate (GTP), and active RHOA interacts downstream with effector molecules ROCK1 and ROCK2 (20). The RHOA/ROCK pathway controls cell migration and other cell functions, mediating the nuclear localization of transcription factors or through direct regulation of the activity of transcription activators by phosphorylation, allowing actin polymerization. MYO9B is a Rho GTPase-activating protein that has four binding sites for myosin light chains, and its function is the local inhibition of RHO activity to enhance directional cell migration (33). Deletion of MYO9B in leukocytes impairs cell migration through increased Rho activity (33).

In sharp contrast, we found that at 24 h of culture, normal fibroblasts induced an increase of the myosin MYO9B, as well as ROCK1 and RHOA, which was associated with an increase of T-cell migration.

The induction of T-cell death and the reduction of its migration capacity may explain the reason why lymphocytes are virtually absent within the FF, indicating that they could not perform this activity. Certainly, other mechanisms may also contribute to the absence of T cells inside FF. For example, it has been shown that fibroblastic foci lack blood vessels, likely due to the epithelial secretion of antiangiogenic factors such as pigment epithelium-derived factor which may also affect T-cell transmigration (34). Likewise, the physical resistance related to the stiffness and aberrant organization of the extracellular matrix may also contribute, as it has been recently suggested in tumor-associated fibrosis (35).

**Figure 8:**
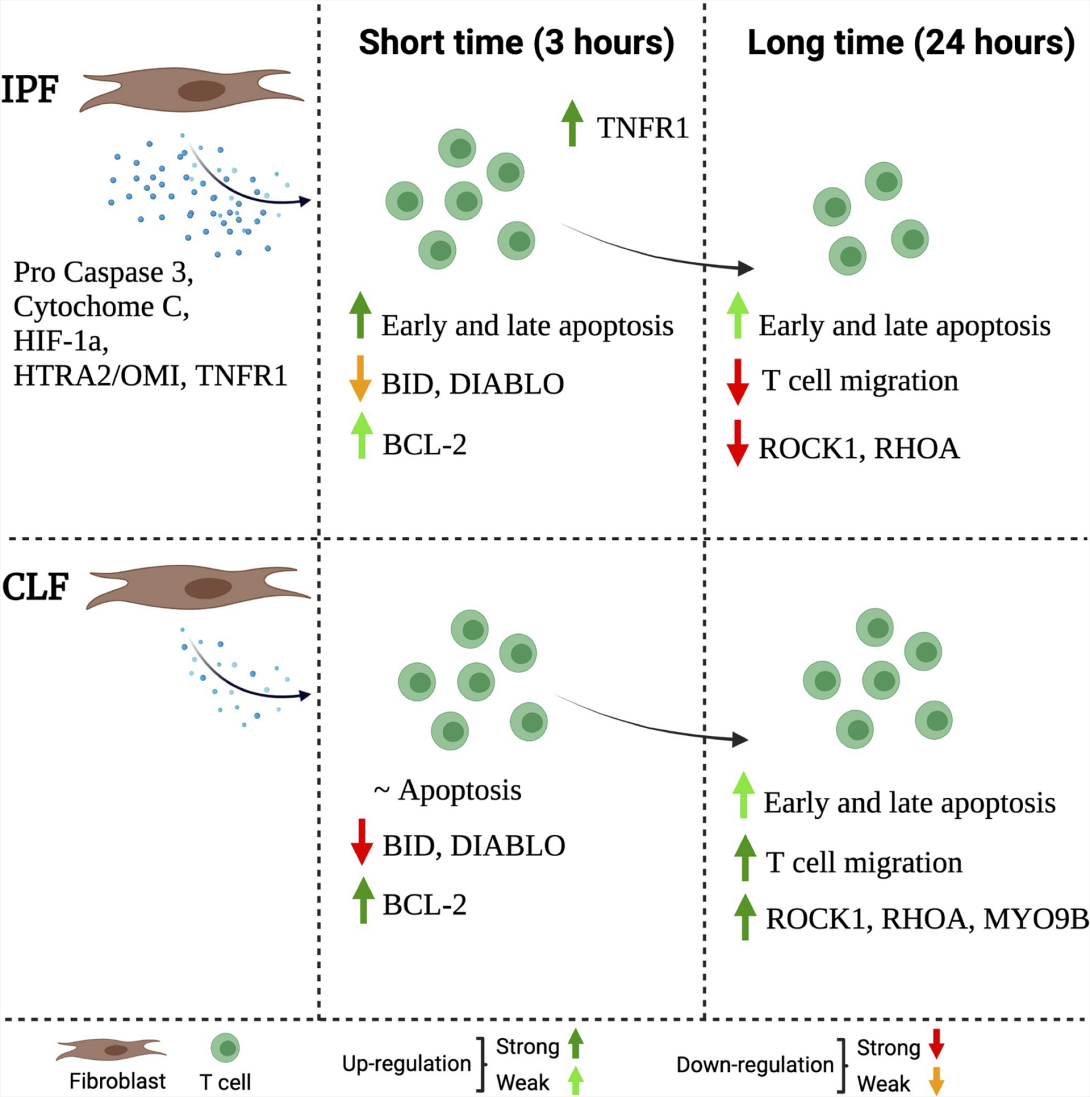
Schematic representation of the proposed mechanism through IPF fibroblasts affects the function of T cells. The IPF fibroblast secretes high levels of pro-apoptotic molecules (left). This microenvironment, at a short time (3 h) of exposure, activates T-cell death, but at the same time, the T cells activate a weak anti-apoptotic profile as a defense mechanism. This profile is characterized by a slight loss of BID and DIABLO as well as a discrete presence of the Bcl-2; moreover, they increase TNFR1 expression (mild). At a long time of exposure (24 h), surviving T cells have decreased the expression of MYO9B, ROCK1, and RHOA, and consequently, these T cells are unable to migrate (right). The figure was created in BioRender.

In summary, we propose a mechanism to explain, at least partially, why the microenvironment generated by IPF fibroblasts affects the T-cell function. IPF fibroblasts secrete high levels of pro-apoptotic molecules such as pro-caspase 3, cytochrome C, HIF-1a, HTRA2-OMI, and TNFR1 (**Figure 8**, left). This microenvironment activates T-cell death after a short time of exposure, despite T cells activating an anti-apoptotic profile as a defense mechanism; this is a weak response, which is characterized by a small loss of BID and DIABLO and a discrete increase of Bcl-2; moreover, they display an increase of TNFR1 expression (**Figure 6**, middle). At long-time exposure, surviving T cells have decreased expression of ROCK1 and RHOA and, consequently, displayed a marked reduction of T-cell migration (**Figure 8**, right).

Certainly, further insight and understanding of the mechanisms that restrict the trafficking of T cells into the fibroblastic foci may help in the development of future therapeutic strategies.

## Data Availability Statement

The original contributions presented in the study are included in the article/**supplementary material**. Further inquiries can be directed to the corresponding authors.

## Ethics Statement

The studies involving human participants were reviewed and approved by the Ethics Committee of Instituto Nacional de Enfermedades Respiratorias Ismael Cosio Villegas (# B33-20). The patients/participants provided their written informed consent to participate in this study.

## Author Contributions

LC-G and MS designed the research and supervised the study. LC-G, IB-R, AP, and MS examined and interpreted the experimental data. LC-G, CB, AR, JC, CR, MM, AS, and LR-L performed the experiments. LC-G and MS wrote the original draft. All authors reviewed, edited, and approved the final manuscript.

## Conflict of Interest

The authors declare that the research was conducted in the absence of any commercial or financial relationships that could be construed as a potential conflict of interest.

## Publisher’s Note

All claims expressed in this article are solely those of the authors and do not necessarily represent those of their affiliated organizations, or those of the publisher, the editors and the reviewers. Any product that may be evaluated in this article, or claim that may be made by its manufacturer, is not guaranteed or endorsed by the publisher.
