# Supplementary material for: Fibroblasts From Idiopathic Pulmonary Fibrosis Induce Apoptosis and Reduce the Migration Capacity of T Lymphocytes
Source: Front Immunol. 2022 Feb 10;13:820347. doi: 10.3389/fimmu.2022.820347 (PMC8866565; doi:10.3389/fimmu.2022.820347)
Supplement: Supplementary file 1 [file DataSheet_1.docx]

Supplementary Material

**Supplementary Figure 1. Evaluation of CD3+ T cells enrichment**. A representative contour plot obtained by the flow cytometric analysis of one healthy donor is shown. (A) The CD3+ fraction, enriched by negative selection immunomagneto was prepared to flow cytometry; cells gate was selected on the base of forward scatter (FSC)/side scatter (SSC), and the expression of CD2, CD3, CD14 and CD19 was evaluated. (B) At the end of the culture, CD3+ T cells were recovered and prepared for flow cytometry. Identification of CD4+ and CD8+ T cells, cell death, expression of TNF pathway molecules, chemokines receptors and exhausted phenotype were evaluated inside each gate.

**Supplementary Figure 2. IPF fibroblasts secrete high concentration of five pro-apoptotic and 2 anti-apoptotic molecules.** Supernatant of fibroblasts from idiopathic pulmonary fibrosis [IPF (SN)] or control lung fibroblasts [CLF (SN)] were recovered until obtaining 360 μg/protein, and then apoptosis-related proteins were measured with a Proteome ProfilerTM kit. (A) Representative membrane of Proteome ProfilerTM from IPF (SN) (up) and CLF (SN) (down). (B) Pixel density obtained by densitometry using online IMAGEJ 1.39c software. Red arrow indicates pro-apoptotic molecules increased in IPF (SN) and green arrow indicates anti-apoptotic molecules increased in CLF (SN). Bars indicate the mean value from two independent experiments and each molecule was evaluated by duplicate.

**Supplementary Figure 3. IPF fibroblasts induces high expression of tmTNF on CD4+ and C8+ T cells.** T cells from healthy donors were cultured during 3 and 24 hours (h) with IPF (SN) or control lung fibroblasts (CFL) SN, and then cells were recovered and prepared to flow cytometry. The expression of tmTNF, tmTNFR1, and tmTNFR2 was evaluated inside the CD4+ T cells and CD8+ T cells gates. Representative contour plot from five independent experiments.

**Supplementary Figure 4. Stimulation of T-cells with IPF or control supernatants did not change cytokine production nor did cause an exhausted phenotype on T cells**. (A) T cells from five different healthy donors were cultured during 3 and 24 hours (h) with IPF (SN) or control lung fibroblasts (CFL) SN. Then, supernatants were recovered and cytokines levels were evaluated by ELISA (to IL-2) and CBA technique for IL-1β, IL-6, IL-10 and IL-12p70. (B and C) T cells from five different healthy donors were cultured 24 h and recovered and prepared to flow cytometry. The expression of CD28, KLR1 and PD-L was evaluated inside the gate of CD4+ and CD8+ T cells.

**Supplementary Figure 5.** **IPF and controls fibroblasts produce similar levels of CCL2.** Supernatants from lung fibroblasts obtained from ten IPF patients and four healthy controls were recovered in different passages (5-9) and the level of the chemokine CCL2 was evaluated by ELISA in duplicate.
